# Characterizing Preferential Adsorption of Phosphate on Binary Sorbents of Goethite and Maghaemite using *in situ* ATR-FTIR and 2D Correlation Spectroscopy

**DOI:** 10.1038/s41598-019-42575-2

**Published:** 2019-04-16

**Authors:** Junho Han, Hee-Myong Ro

**Affiliations:** Department of Agricultural Biotechnology and Research Institute of Agriculture and Life Sciences, Seoul National University, Seoul, 08826 Republic of Korea

## Abstract

Recent developments in analytics using infrared spectroscopy have enabled us to identify the adsorption mechanism at interfaces, but such methods are applicable only for simple systems. In this study, the preferential adsorption of phosphate on binary goethite and maghaemite was investigated. As a result, monodentate and bidentate complexes were the major complexes on goethite and maghaemite, respectively. A shrinking effect in goethite and a swelling effect in maghaemite were identified, and environmental perturbations caused a significant decrease in the integrated absorbance of phosphate complexes on maghaemite, while no effect was observed on goethite, which implies that different adsorption mechanisms were involved. Based on the results, a bridging complex was proposed, and the swelling effect is explained by the negatively charged maghaemite surface resulting from the bidentate complex. The isolation of phosphate by the shrinking effect explains the low phosphate bioavailability in the soil environment, while the colloidal properties of the bidentate complex on maghaemite are the reason for colloidal mobilization. To the best of our knowledge, this study not only addresses the shrinking and swelling properties of iron (hydr)oxide nanoparticles but also demonstrates preferential adsorption on binary sorbents using *in situ* ATR-FTIR for the first time.

## Introduction

Phosphorus is one of the essential elements in the soil environment and is a key element for the growth of terrestrial organisms in most environments. In the past, phosphorus acted as a limiting factor for cultivation; however, recent developments in fertilization have caused over-fertilization in most agricultural regions to maximize agricultural productivity^1^. As a consequence, excess phosphorus has run off into the water system, leading to eutrophication and creating serious health and environmental problems worldwide^1–3^. Therefore, to retain phosphorus in the soil environment, it is essential to understand how phosphorus interacts with soil components and what critical factor governs the structural configuration of phosphorus^4,5^.

Numerous studies have been conducted to identify the adsorption mechanisms of phosphate (the oxyanion form of phosphorus) on individual soil components or soil mixtures^6–12^. Among the various technical and experimental schemes, *in situ* attenuated total reflection-Fourier transform infrared spectroscopy (ATR-FTIR) has shown interesting results for interpreting the adsorption mechanism of phosphate by utilizing the evanescent wave from the total reflection at the interface between the ATR crystal and singly formed metal (hydr)oxide in the aqueous phase. The weakness of ATR-FTIR has historically been the complex overlapping peaks resulting from the various vibrations of molecular structures and is a major problem for data interpretation; however, recent developments in computational chemistry have enabled us to separate overlapping peaks and to identify the structural configuration of complexes between oxyanions and metal (hydr)oxides^4,12,13^.

The developed technologies are not satisfactory for interpreting adsorption mechanisms in the real environment because *in situ* ATR-FTIR with computational chemistry is available only for simple systems, for example, one or two solutes on a single sorbent. Many studies have identified the structural configuration of phosphate by changing the environmental conditions (pH, ionic strength and surface loading)^11,14,15^, and a few studies have already examined the competitive adsorption of several oxyanions onto a single metal (hydr)oxide^14,16–18^. However, no articles were found employing dual or multiple sorbents in *in situ* ATR-FTIR that could interpret the preferential adsorption of oxyanions onto the target sorbents.

For that reason, we have attempted to characterize preferential adsorption on binary sorbents using *in situ* ATR-FTIR. Phosphate was employed as a target solute, and goethite (Gt) and maghaemite (Mh), which showed significantly different spectra in our preliminary experiment and relatively high stability, were selected as the binary sorbents. Previous studies have also identified the spectral change with respect to surface loading^4,19,20^; thus, the high surface loading condition (0.67 mmol g^−1^) from the preliminary experiment was employed. This condition resulted in no significant changes in the spectra that would demonstrate preferential adsorption on the binary sorbents using *in situ* ATR-FTIR. In this study, a procedure was developed and demonstrated to characterize preferential adsorption using *in situ* ATR-FTIR on binary sorbents. Semi-quantification was conducted to calculate the fraction of preferential adsorption of phosphate between the binary sorbents, and shrinking, swelling and dehydration effects were addressed to understand the dynamics between phosphate and the binary sorbents.

## Results and Discussion

### Physicochemical characteristics and adsorption isotherm

The physicochemical characteristics of the purchased Gt and Mh were previously identified and reported (Table S1)^5,21^. Briefly, Gt and Mh were identified as rod- and spherical-shaped nanoparticles (NPs) with sizes of 50.3 × 10.8 nm and 50.7 nm, respectively, using high-resolution transmission electron microscopy (HR-TEM). The point of net zero charge and surface area (SA) were measured as 5.7 and 84.0 m^2^ g^−1^ for Gt and 4.7 and 35.6 m^2^ g^−1^ for Gt, respectively. The theoretical SA was calculated based on the measured size using HR-TEM and cell volume data from the literature^22^. The results were 113.1 and 23.0 m^2^ g^−1^ for Gt and Mh, respectively. Rectangular and spherical shapes were assumed for the calculation, and the average size of NPs was used; thus, it was difficult to correlate the measured SA and calculated SA. However, the trend was compared, and opposite patterns were found in the measured SA and calculated SA. The calculated SA was higher than the measured SA for Gt, but the opposite pattern was observed for Mh. Based on field emission scanning electron microscopy/energy-dispersive spectroscopy (FE-SEM/EDS) and HR-TEM observations, a more densely aggregated structure was found in Gt than in Mh, and the rod-shaped Gt NPs were oriented side by side, which possibly decreased the available surface area by blocking the adsorption of the N_2_ sorbate during BET measurements. However, the spherical shape of Mh minimized the decrease in SA due to the contact between NPs, and it was difficult to identify small NPs (<10 nm) and calculate an average diameter; thus, the measured SA might be higher than the calculated SA. The X-ray diffraction (XRD) (Fig. S1) and HR-TEM (Fig. S2) results clearly confirmed the structure of the NPs as Gt and Mh based on the American Mineralogist Crystal Structure Database (AMCSD).

The maximum adsorption capacity (*Q*_*max*_) of Gt and Mh from the Langmuir isotherm were previously reported as 0.352 and 0.296 mmol g^−1^, respectively, at pH 4, and the Langmuir constant (*K*_*L*_) values of Gt and Mh were calculated as 8.93 and 2.13 L g^−1^ at pH 4. Gt showed higher *Q*_*max*_ and *K*_*L*_ values than Mh. In the previous literature, several studies have already reported the adsorption capacity of Gt^23–26^ (Fig. S3), but no study was found for Mh. The difference in adsorption capacity (*Q*) between suspended NPs and the corresponding film was also assessed. The *Q* of suspended NPs with agitation in a 1.1 mM P solution at pH 4 was 0.202 and 0.145 mmol g^−1^ for Gt and Mh, while the *Q* of the film was 0.157 and 0.134 mmol g^−1^ for Gt and Mh, respectively. The difference between the two batch experiments was 0.045 and 0.011 mmol g^−1^ for Gt and Mh, respectively. This difference clearly indicates the decrease in adsorption capacity resulting from film formation; this decrease might be caused by side-by-side aggregation of nano- and rod-shaped Gt, which decreases the number of adsorption sites. Based on the Langmuir isotherm and difference in *Q* resulting from film formation, it was presumed that the difference in *Q*_*max*_ between Gt and Mh and in *Q* between the NP suspensions and films could be attributed not only to the difference in binding affinity on the surface but also to the difference in NP aggregation *via* phosphate bridging^27^. The effect of phosphate on colloidal NPs was assessed in a sedimentation experiment (Fig. S4). Increasing phosphate concentration accelerated sedimentation on Gt and Mh; a less obvious result was observed for Gt, while a clear result was found for Mh. This difference occurred because the majority of Gt NPs immediately settled in all controls and treatments, while the minority of Gt NPs showed colloidal properties in the absence of phosphate. The results clearly indicated that Gt and Mh aggregates were both related to the presence of phosphate but that aggregation in Gt NPs was not only caused by phosphate.

### Morphology and abundance of binary sorbents

Based on FE-SEM observations, the Mh film had a relatively uniform thickness on the ZnSe crystal, while a few μm-sized aggregates were observed in the Gt layer, which appeared less uniform in thickness. The layer thicknesses of Gt and Mh were approximately 1.5 and 1.1 μm, respectively. The aggregates in the Gt film had diameters of up to 6 μm. High-magnification FE-SEM and HR-TEM images clearly confirmed that the μm-sized particles were aggregates of Gt nanorods (Figs S2 and S5). The difference in uniformity between the two sorbents was explained by the sedimentation experiment, which showed faster settlement in Gt than in Mh without phosphate, which indicates that the Gt NPs were more aggregated; thus, aggregation caused less uniformity in the film formation process. The decrease in the pH of the Gt dispersion below 2 led to a dramatic decrease in aggregation, while sonication was not effective. The pH was controlled to 4 to prevent damage to the ZnSe crystal and to meet our target pH condition for the experiment.

**Figure 1:**
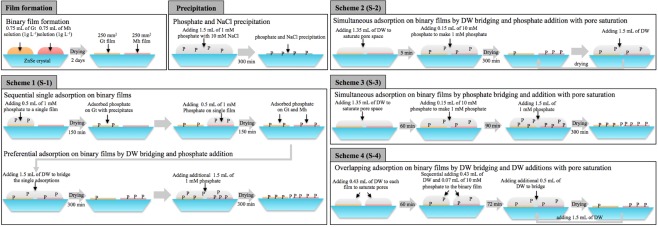
Schematic diagram of film formation, phosphate precipitation with NaCl, single adsorption and four experimental schemes in this study. Single adsorption is the reaction of one sorbent with overlaid water, and simultaneous adsorption is the reaction of two binary sorbents with overlaid and bridged water. Three different pre-saturation conditions were set up: dehydrated, hydrated with DW for 5 min and hydrated with DW for 60 min, to evaluate the swelling properties of iron (hydr)oxides. After adsorption experiments on binary sorbents with bridging, the samples were dehydrated or remained hydrated, and additional DW or phosphate solution was applied to identify the adsorption change.

Four experimental schemes were applied in this study (Fig. 1), and it was essential to maintain the sorbent concentration during the experiment. For that reason, the peak areas (PAs) of two distinctive regions (840-760 cm^−1^ for Gt and 760-670 cm^−1^ for Mh) were calculated and are summarized in Table S2 and Fig. S6. The ratios between the two PAs (Gt/Mh) of S-1, S-2, S-3 and S-4 after film formation were 0.654, 0.703, 0.665 and 0.695, which showed similar values, and the ratios before and after phosphate adsorption were 0.963, 0.988, 1.152 and 1.038, respectively (Table S2). The PAs of Mh were relatively constant, while the PAs of Gt slightly increased, but the phosphate peak at 1300-900 cm^−1^ might increase the background of the Gt peak at 840-760 cm^−1^; thus, it is hard to conclude whether aggregation occurs when the ratio increases. In addition, there is a possibility that more Gt or Mh NPs aggregated in the evanescent wave region during the experiment and caused the increase in background.

Semi-quantification using FE-SEM/EDS was also conducted, and the atomic ratio (P/Fe) was 0.0521 on Gt and 0.0656 on Mh. For each sorbent, 0.75 mg was analysed, and the Fe concentration of Gt (FeOOH) and Mh (Fe_2.67_O_4_) was 1.125 and 1.253 mmol g^−1^; thus, the adsorbed phosphate content was 0.44 and 0.62 μmol for 0. 75 mg of Gt and Mh, respectively. No precipitate was detected on the ZnSe crystal, and it was presumed that the solubility of sodium phosphate (118 g L^−1^) was higher than the experimental conditions (0.156 g L^−1^). Regarding the effect of dehydration, charged phosphate precipitated on the sorbent surface, where water molecules were ultimately retained. In addition, no significant difference was found in the P/Fe atomic ratio between the margin and centre of the layers in all experiments (data not shown). However, a gradient distribution in NaCl precipitation was found: the concentration and size of NaCl precipitates were higher at the margin of the layer and gradually decreased at the centre (Fig. S7). The amount of adsorbed phosphate on Mh was 1.4 times as high as that on Gt, and this result was similar to the results of the ATR-FTIR experiment. In addition, 1 μmol of phosphate was introduced, and 1.06 μmol was measured by FE-SEM/EDS analysis; thus, the recovery was 106%, which was a satisfactory result considering the relatively high error of FE-SEM/EDS measurements.

### ATR-FTIR spectra of initial single or simultaneous adsorption

**Figure 2:**
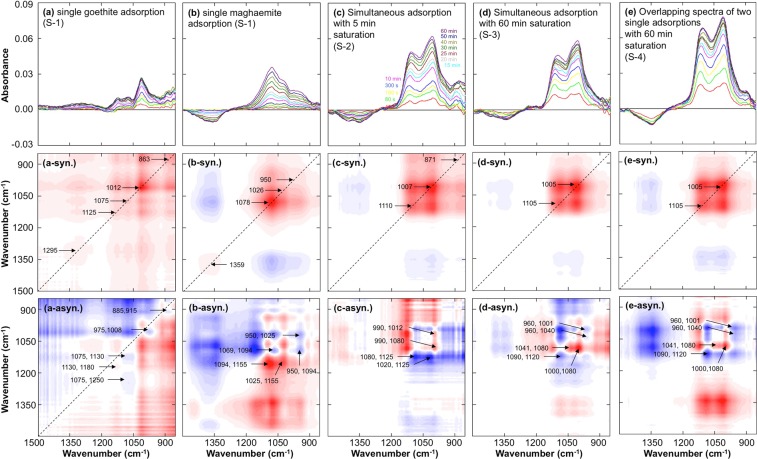
The five top graphs show the evolution of the time series of ATR-FTIR spectra of 1 mM phosphate on single Gt (**a**) and single Mh (**b**), simultaneous adsorption on binary sorbents with 5 min saturation (**c**), simultaneous adsorption on binary sorbents with 60 min saturation (**d**), and overlapping spectra of two single adsorptions with 60 min saturation (**e**). The spectra were calculated by subtracting the spectra at each time from the initial spectra after water saturation. The five middle graphs and bottom graphs are the synchronous (suffix of syn.) and asynchronous contour plots (suffix of asyn.), respectively, corresponding to the top graphs. The numbers in the middle and bottom graphs indicate autopeaks and crosspeaks, respectively.

The time series of spectra for initial phosphate adsorption on single and binary sorbents were plotted, and synchronous and asynchronous plots were illustrated by using two-dimensional correlation spectroscopy (2D-COS) (Fig. 2). In single adsorption on Gt (S-1), the peaks at 1295, 1125, 1075, 1012, and 863 cm^−1^ were identified as autopeaks in the synchronous plot (Fig. 2a-syn.), and crosspeaks at 1180, 975, 915, and 885 cm^−1^ were observed in the asynchronous plot (Fig. 2a-asyn.). For single adsorption on Mh (S-1), the peaks at 1359, 1078, 1026 and 950 cm^−1^ were identified as autopeaks (Fig. 2b-syn.), and crosspeaks at 1155, 1094, and 1069 cm^−1^ were observed (Fig. 2b-asyn.). Simultaneous adsorption on the binary sorbents with pre-saturation (S-2 for 5 min and S-3 for 60 min) using distilled water (DW) is illustrated in Fig. 2c,d, and the overlapping spectra from two single adsorptions on Gt and Mh (S-4) are plotted in Fig. 2e. The synchronous and asynchronous plots are placed below the graphs. The simultaneous adsorption spectra and overlapping spectra of two single adsorptions showed similar peak positions: 1105 and 1005 cm^−1^ as the autopeaks and 1120, 1090, 1080, 1040, 1000 and 960 cm^−1^ as the crosspeaks. The peak positions in Fig. 2c slightly differed from those in Fig. 2d,e, and there was a significant increase at low wavenumbers (950-850 cm^−1^), which was not observed in S-3 and S-4, where DW pre-saturation for 60 min was employed.

Based on the previous literature, a high surface loading was set in this study (23.8 and 56.2 mmol m^−2^ for Gt and Mh, respectively) to minimize changes in surface complexes during adsorption^19,21^. The results for phosphate adsorption on a single sorbent showed a distinctive spectrum compared with previous studies^12,28–30^: the background increased at 1500-1200 and 930-850 cm^−1^ for Gt^29,30^ (Fig. 2a) and decreased at 1500-1300 cm^−1^ for Mh (Fig. 2b). For S-1, spectra were recorded by overlaying 1 mM phosphate solution on the dried Gt; thus, there were possible fluctuations in absorbance due to the Gt sorbent. For this reason, pre-saturation with DW was conducted before the phosphate adsorption experiment (5 min pre-saturation in S-2 and 60 min pre-saturation in S-3 and S-4); a background increase was found in the dried sample and the sample with 5 min pre-saturation (S-1 and S-2), while no background increase was observed in the samples with 60 min pre-saturation (S-3 and S-4). Based on this observation, the nanopores in Gt require more time to saturate with water, which causes a dramatic increase in absorbance at low wavenumbers. For single adsorption on Mh, a decrease in the broad peak at 1450-1300 cm^−1^ was observed. It was initially assumed that the desorption of surface carbonate occurred by competition with phosphate, but later studies confirmed that the decrease in Mh concentration in the evanescent wave path was the reason for the decrease in the broad peak at 1450-1300 cm^−1^: this broad peak was still observed under argon gas purging at a flux of 1 L min^−1^ from film formation to the end of the experiment. Unlike the slow water saturation in Gt, Mh showed no significant increase at 930-850 cm^−1^, which implies that water molecules readily saturated into the relatively large pores of Mh.

Distinctive adsorption patterns were observed between Gt and Mh. The peaks at 1012 and 1078 cm^−1^ showed the highest absorbance in single Gt adsorption and single Mh adsorption, respectively. The absorbance increase was plotted as a time series (Fig. S8), and the linear correlation coefficients among the peaks were calculated (Tables S3 and S4). As a result, the relationship between absorbance and time for phosphate on Gt and Mh showed a distinctive pattern of non-linear and linear curves, respectively. The peaks in phosphate-adsorbed Gt (P-Gt) were categorized into three groups: low wavenumbers (863, 885 and 915 cm^−1^, r^2^ > 0.939), phosphate adsorption peaks (975, 1012, 1075 and 1125 cm^−1^, r^2^ > 0.891), and high wavenumbers (1180 and 1295 cm^−1^, r^2^ = 0.944). As discussed above, the peaks in the low-wavenumber region were attributed to the slow seepage velocity of water molecules into the nanopores of Gt, which caused a gradual increase in water over time. The peaks in the high-wavenumber region might be caused by increased background absorbance or the aggregation of Gt. However, the phosphate adsorption peaks of Gt and Mh (except the peak at 1359 cm^−1^) showed a linear and significant correlation (r^2^ > 0.891 for Gt and r^2^ > 0.933 for Mh), which indicates that a consistent adsorption mechanism was involved in phosphate adsorption on Gt and Mh.

The structural configuration of phosphate complexes on Gt has been widely studied for several decades, but the results are still controversial^4,9,11,15,16,18,19,28–30^. Atkinson *et al*. first reported the bidentate binuclear (BB) complex in 1974, and numerous studies have identified the BB complex as the major structural configuration on iron (hydr)oxides; however, recent studies have confirmed changes in structural configuration due to surface loading and pH conditions, in which the BB complex was dominant at low surface loadings, while the monodenate mononuclear (MM) complex was abundant at high surface loadings, as determined by extended X-ray absorption fine structure spectroscopy^19^. In addition, the diprotonated monodentate mononuclear complex (MMH_2_) and monoprotonated MM complex (MMH_1_) were identified as major complexes at high surface loading conditions using the infrared surface titration technique with the CD-MUSIC model^29^, and MMH_2_, monoprotonated BB (BBH_1_) and nonprotonated BB (BBH_0_) were observed in an ATR-FTIR study with density functional theory (DFT) calculations^30^. In addition, Kubicki *et al*. stated that MM, BB and outer-sphere complexes occur together depending on the adsorbent surface and environmental conditions^12^. Based on the DFT calculations and ATR-FTIR study of Yang *et al*.^30^, MMH_2_ (1125 and 1012 cm^−1^) and MMH_1_ (975 and 1075 cm^−1^) complexes were identified in Gt in this study, and MMH_2_ was found as the most abundant phosphate complex on Gt. In addition, BBH_1_ (1150, 1078 and 950 cm^−1^) and BBH_0_ (1094, 1026 and 950 cm^−1^) complexes were confirmed in phosphate-adsorbed Mh (P-Mh), but it was impossible to identify the most abundant complex in Mh because of the complex overlapping spectra. Unlike Gt, phosphate complexes on Mh were not previously reported, but a distinctive spectrum was found, implying that different complexes are dominant on Gt and Mh, i.e., monodentate complexes for Gt and bidentate complexes for Mh.

### ATR-FTIR spectra for subsequent bridging and addition of DW and phosphate

**Figure 3:**
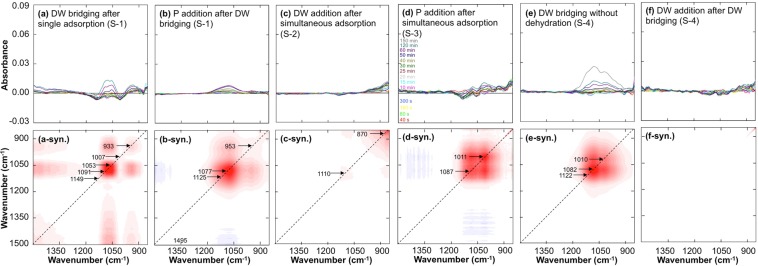
The six top graphs show the evolution of the time series of ATR-FTIR spectra of phosphate after sequential DW bridging and phosphate addition in S-1 (**a**,**b**), DW and phosphate addition after simultaneous adsorption on binary sorbents in S-2 and S-3 (**c**,**d**), and DW bridging without dehydration and DW addition after dehydration in S-4 (**e**,**f**). The spectra were calculated by subtracting the spectra at each time from the initial spectra after water saturation. The lower six graphs show the synchronous plots calculated by 2D-COS (suffix of syn.). The numbers indicate autopeaks.

The time series of spectra for the subsequent bridging and addition of DW and phosphate after single and simultaneous adsorption were plotted, and synchronous plots were illustrated by 2D-COS (Fig. 3). The spectra were manipulated by subtracting the initial spectrum after water saturation (120 sec after solution input without DW pre-saturation). In S-1, single adsorption on Gt or Mh was first conducted, and the samples were fully dried; then, DW bridging and phosphate addition were conducted sequentially without drying. As a result of DW bridging, the peaks at 1150 and 1010 cm^−1^ significantly decreased initially, and the peak at 1075 cm^−1^ increased later (Fig. 3a). After 300 min, additional phosphate solution (0.2 mL of 10 mM) was added to the dehydrated sample, and the broad peak centred at 1077 cm^−1^ was elevated (Fig. 3b). In S-2, simultaneous adsorption on the binary sorbents was first conducted, and the samples were fully dried; then, DW addition was conducted. A significant background increase at low wavenumbers (1000-850 cm^−1^) was observed with no peak appearance (Fig. 3c). This behaviour implies that there was water saturation at a low seepage velocity during adsorption and that no additional preferential adsorption occurred. S-3 followed a similar procedure to that of S-2, but phosphate addition was employed instead of DW addition, and the peaks at 1078 and 1011 cm^−1^ increased with increased background absorbance at low wavenumbers (950-850 cm^−1^) (Fig. 3d), which implies that P-Gt was increased compared to P-Mh due to additional phosphate loading. The peaks in S-3 differed from the broad peak centred at 1077 cm^−1^ in S-1; the same phosphate addition was applied, but dehydration was applied in S-1 but not in S-3. The reason for the opposite pattern is discussed below. In S-4, single adsorption on Gt and Mh with 60 min DW pre-saturation was conducted, and DW bridging was employed without dehydration. After DW bridging, the binary sorbents were dried, and additional DW was applied. As a result, broadly overlapping peaks at 1122, 1082 and 1010 cm^−1^ were identified, and no fluctuation at low wavenumbers was observed (Fig. 3e). After DW addition, no significant peak was observed, and only a slight background increase was identified at low wavenumbers. The background increase at low wavenumbers was only observed after drying the samples, which indicates the effect of water saturation during the experiment.

### Linear least squares regression of ATR-FTIR spectra

**Figure 4:**
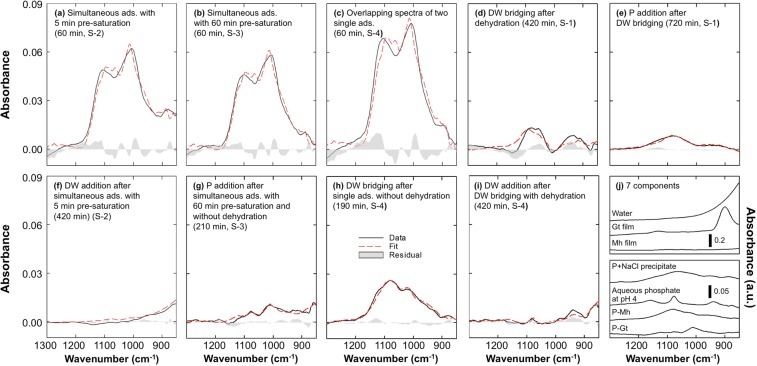
LLS regression results for initial simultaneous (**a**,**b**) and separate adsorption (**c**) using spectra recorded 60 min after phosphate input, and later adsorption changes after DW bridging in S-1 (**d**), P addition in S-1 (**e**), DW addition in S-2 (**f**), P addition in S-3 (**g**), DW bridging in S-4 (**h**) and DW addition in S-4 (**i**); the recording time for each spectrum is marked in each graph. The graph on the bottom left shows the 7 components employed in the LLS regression.

The ATR-FTIR spectra of adsorption after phosphate input and the spectra after bridging and the addition of DW and phosphate were collected, and the fitting results of linear least squares (LLS) regression with 6 or 7 parameters were plotted and summarized (Fig. 4 and Table 1). The spectra after 60 min of phosphate input were significantly similar except for an increase at low wavenumbers in S-2 (Fig. 4a) and elevated absorbance in S-4 compared with the others (Fig. 4b). As discussed earlier, the slow water seepage velocity caused the increase. Different concentrations of phosphate were applied (1.5 mL of 1 mM for simultaneous adsorption in S-2 and S-3 and 1.0 mL of 1.5 mM for overlapping single adsorptions in S-4 to match the final concentration), and no significant difference in the absorbance of the two sorbents was observed for all schemes. Thus, it was concluded that the difference in initial concentration caused the elevated absorbance in S-4. The coefficients of P-Gt were 1.794, 1.682, and 2.100, while the coefficients of P-Mh were 1.433, 1.393 and 1.938 for S-2, S-3, and S-4, respectively. The coefficient ratios (P-Gt/P-Mh) were 1.25, 1.21 and 1.08 for S-2, S-3 and S-4, respectively, and the ratio for S-4 was significantly different from those of S-2 and S-3, where no preferential adsorption occurred and a higher concentration was initially applied.

**Table 1:** Linear least squares (LLS) regression results calculated with 7 components (spectra of single phosphate adsorption on goethite (P-Gt) and maghaemite (P-Mh), water, aqueous 1 mM phosphate at pH 4 (P-Aq.), goethite (Gt) and maghaemite (Mh) films, and precipitated phosphate with NaCl (P pre.)) for the adsorption experiment.

Spectral data used for regression			Regression parameters		Results of linear least squares regression							
Scheme	Time (min)	Description	adj. R^2^	Std. err.		P-Gt.	P-Mh.	Water	P-Aq.	Gt. film	Mh. film	P pre.
S-2	60	Simultaneous ads. with 5 min pre-saturation	0.9726	0.0034	Coefficient	1.794	1.433	0.016	0.000	0.012	−0.114	—
					Std. err.	0.042	0.027	0.002	0.000	0.002	0.008	—
S-3	60	Simultaneous ads. with 60 min pre-saturation	0.9794	0.0029	Coefficient	1.682	1.393	−0.016	0.000	0.010	−0.101	—
					Std. err.	0.036	0.023	0.002	0.000	0.005	−0.017	—
S-4	60	Two overlapping single ads. with 60 min pre-saturation	0.9658	0.0051	Coefficient	2.100	1.938	−0.038	0.000	0.019	−0.145	—
					Std. err.	0.063	0.041	0.003	0.058	0.004	0.013	—
S-1	420	DW bridging after dehydration	0.6266	0.0027	Coefficient	−0.320	0.631	0.004	−0.056	0.005	0.143	−0.249
					Std. err.	0.044	0.043	0.002	0.033	0.002	0.029	0.053
S-1	720	P addition after DW bridging	0.9654	0.0005	Coefficient	−0.122	0.247	−0.004	0.006	0.003	0.001	0.013
					Std. err.	0.008	0.008	0.000	0.006	0.000	0.005	0.010
S-2	420	DW addition after simultaneous ads.	0.9680	0.0019	Coefficient	0.057	0.038	0.024	0.026	0.001	−0.005	−0.031
					Std. err.	0.010	0.010	0.000	0.008	0.001	0.007	0.012
S-3	210	P addition after simultaneous ads.	0.8912	0.0012	Coefficient	0.421	0.059	0.016	0.032	−0.002	0.007	−0.037
					Std. err.	0.019	0.019	0.001	0.015	0.001	0.013	0.024
S-4	222	DW bridging after single ads. without dehydration	0.9867	0.0010	Coefficient	0.209	0.749	−0.006	−0.081	−0.002	−0.010	0.008
					Std. err.	0.016	0.016	0.001	0.012	0.001	0.011	0.020
S-4	420	DW addition after DW bridging with dehydration	0.8684	0.0011	Coefficient	0.069	−0.037	0.025	0.163	−0.009	0.050	−0.084
					Std. err.	0.018	0.018	0.001	0.014	0.001	0.012	0.022

Precipitated phosphate was not employed for the LLS regression with pre-saturation (60 min). The columns adj. r^2^ and Std. err. indicate the adjusted coefficient of determination and standard error of estimate, respectively. The time indicates the recorded time after initial phosphate addition.

In S-1, DW bridging was employed in single adsorption on binary sorbents after 300 min, and the spectrum recorded at 420 min was subtracted from the spectrum at 302 min (Fig. 4d). After DW bridging, the sample was dried, and phosphate solution was added (Fig. 4e). LLS regression with 7 components was conducted, and the coefficients of P-Gt and P-Mh were −0.320 and 0.631, respectively, for DW bridging and −0.122 and 0.247 for phosphate addition. The results implied that the phosphate content on the Gt surface was decreased while that on Mh was increased, which indicates the occurrence of preferential adsorption on Mh over Gt. However, there was a relatively poor fit result (adjusted correlation coefficient (adj. r^2^) = 0.627), and the significance level of the Mh film was calculated, which could not explain the increase in the Mh film while the Gt film was constant. For that reason, experiments S-2, S-3 and S-4 were conducted.

In S-2 and S-3, simultaneous adsorption on the binary sorbents was measured, and additional DW after full dehydration of the sample was applied for S-2 (Fig. 4f), while phosphate addition without dehydration was applied for S-3 (Fig. 4g). As a result, no significant components were observed after DW addition in S-2, which implies that the complex in the sample system reached equilibrium before applying additional DW. In addition, the increase at low wavenumbers confirmed the low seepage velocity of water (Fig. 4f). The coefficient of P-Gt (0.421) in S-3 was significantly higher than the coefficient of P-Mh (0.059) (Fig. 4g). This difference indicates that additional phosphate was adsorbed with Gt, while no additional complex on Mh was found. This result contrasted with P addition in S-1.

In S-4, overlapping single adsorptions were conducted, and DW bridging was applied 72 min after the initial phosphate input; then, dehydration and DW addition were sequentially employed. The spectrum for DW bridging recorded at 190 min was subtracted from the spectrum at 74 min (Fig. 4h), and the spectrum for DW addition recorded at 420 min was subtracted from the spectrum at 302 min. LLS regression was applied with 7 components. As a result, P-Gt and P-Mh were 0.209 and 0.749 for DW bridging and 0.069 and −0.037 for DW addition, respectively. This result indicates that more phosphate was adsorbed onto the Mh surface than onto the Gt surface. No significant peak was observed with DW addition, which indicates that the preferential adsorption reached equilibrium.

### LLS regression for time series measurements

**Figure 5:**
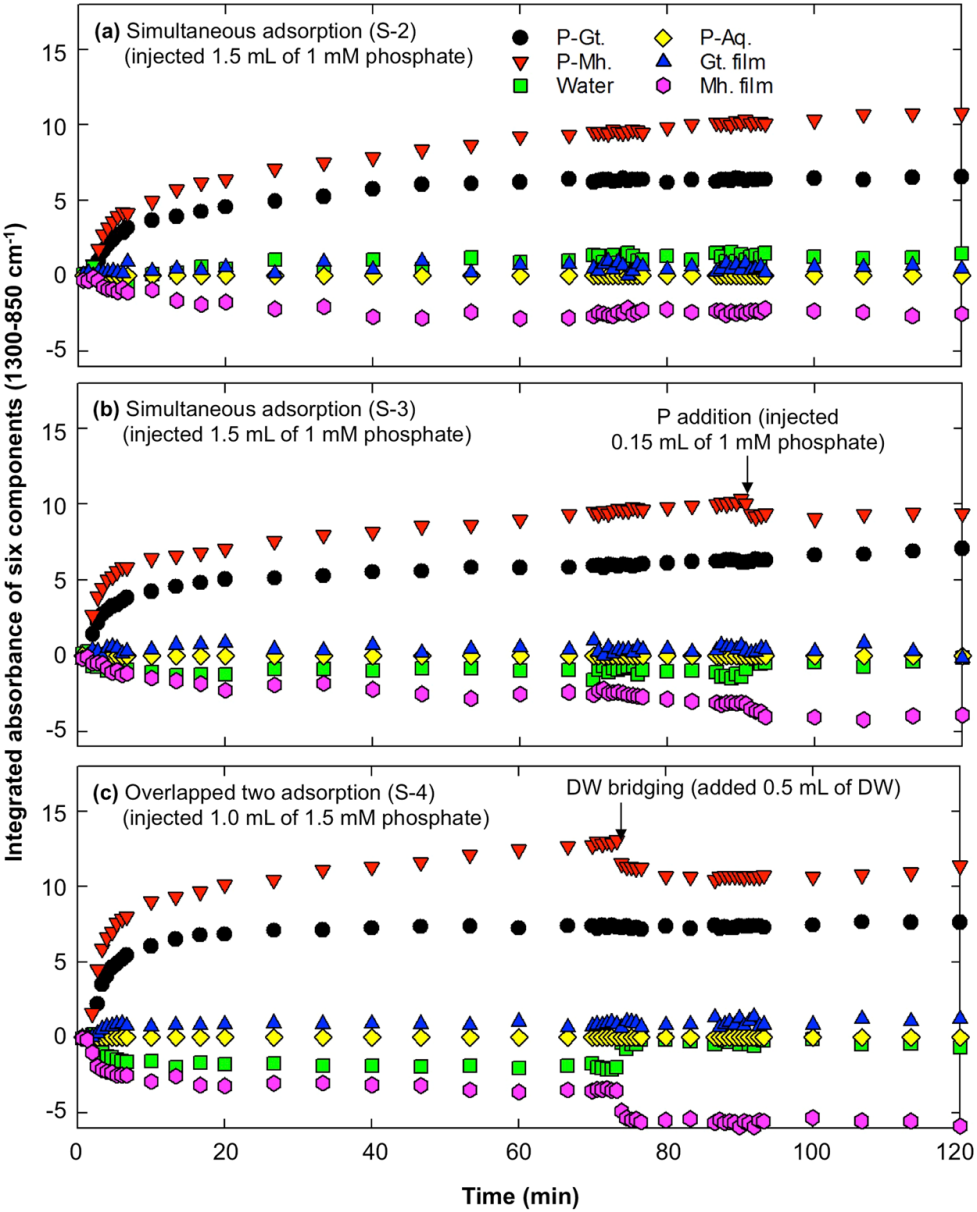
Time series scatter plots of integrated absorbance calculated by LLS regression in simultaneous adsorption with 5 min pre-saturation (S-2), simultaneous adsorption with 60 min pre-saturation and phosphate addition (S-3), and overlapping two single adsorptions with 60 min pre-saturation and DW bridging (S-4). P-Gt. and P-Mh. indicate the spectra from single adsorption after 60 min in S-1; water and P-Aq. indicate the spectra of water background and aqueous phosphate at pH 4, and Gt. and Mh. films are the spectra of dried Gt and Mh, respectively.

The experimental results and LLS regression clearly confirmed the presence of preferential adsorption, but there was still a controversial result in this experiment (opposite patterns after phosphate addition in S-1 and S-3), and the components were not normalized for the LLS regression; thus, it was difficult to compare the contribution of each component to the spectrum. For that reason, we conducted LLS regression for 120 min of data for S-2, S-3 and S-4, and the results of the LLS regression are plotted in Fig. 5. The integrated absorbance (coefficient x PA of each component, hereafter IA) was employed instead of the coefficient to compare the contribution of each component to the absorbance, and precipitated phosphate was excluded because no significant presence was identified in the previous LLS regression (Table 1); thus, 6 components were employed.

The IA of water was increased in S-2 but decreased in S-3 and S-4, where 60 min DW pre-saturation was applied. In addition, P addition in S-3 and DW bridging in S-4 showed a dramatic increase in the IA of water, while IA was constant in S-2. The IA of aqueous phosphate was 0.006–0.047 at 40 sec but approached zero (<10^−6^) over time, which indicates that the aqueous and outer-sphere complexes of phosphate were readily adsorbed onto the iron (hydr)oxide surfaces; however, the adj. R^2^ of the LLS regression at the initial time (<0.5) was significantly lower than the later (>0.9). The IA of the Mh film dramatically decreased over time, while the IA of the Gt film slightly increased over time. The average IA of the Gt and Mh films for the 120 min was 0.47 and −2.01 for S-2, 0.40 and −2.14 for S-3, and 0.82 and −3.98 for S-4, respectively. This result indicates that the Gt concentration increased in the evanescent wave region and the phosphate adsorbed on Gt also increased. Unlike Gt, the opposite pattern was observed in Mh. Based on the results, a shrinking effect on Gt and swelling effect on Mh were observed; these effects played an important role not only in the stability of the film but also in phosphate adsorption.

The IAs of P-Gt and P-Mh at 60 min were 6.19 and 9.22 for S-2, 5.80 and 8.96 for S-3, and 7.25 and 12.47 for S-4, respectively. The IAs of P-Gt and P-Mh at 120 min were 6.56 and 10.8 for S-2, 7.06 and 9.37 for S-3, and 7.63 and 11.4 for S-4, respectively. The IA ratios (Gt/Mh) of S-2, S-3 and S-4 at 60 min were 0.67, 0.65 and 0.58, respectively, while the ratios at 120 min were 0.61, 0.75 and 0.67. The IA of P-Mh was higher than the IA of P-Gt, but P-Gt showed no significant fluctuation with P addition or DW bridging, while P-Mh showed a significant decrease. It was presumed that the decrease in Mh concentration in the evanescent wave region decreased the IA of P-Mh. In addition, the ratios were significantly different. The ratios of IA at 60 min in S-2 and S-3 showed similar values and were also similar to the ratio of IA at 120 min in S-4, which represented DW bridging. The ratio of IA was changed by environmental perturbations (adsorption time, DW bridging or P addition).

### Shrinking and swelling due to phosphate complexes

Based on the LLS regression of the time series measurements, shrinking and swelling properties were identified, which decreased the IA of P-Mh and the Mh film and changed the IA of water in the evanescent wave region. However, these properties were insufficient to explain the opposite patterns after phosphate addition in S-1 and S-3. In addition, peak assignment from previous DFT calculations identified MMH_1_ and MMH_2_ complexes for P-Gt and BBH_0_ and BBH_1_ complexes for P-Mh^4,29,30^; thus, the monodentate complex of Gt should be more susceptible to environmental perturbations than the bidentate complex of Mh^13,31^, but the opposite trend was observed in this study. Furthermore, the phosphate-adsorbed spectrum of Gt in this study was significantly different from those in previous studies^8,30^, but a similar spectrum was found^29^. Based on these results, we hypothesized the formation of a bridging complex on Gt.

The reasons for this hypothesis are as follows: 1) the difference in spectral results compared with previous studies, 2) the thermodynamic stability of monodentate and bidentate complexes, 3) the decrease in water seepage velocity with repeated phosphate addition, and 4) the stability of phosphate complexes on the Gt surface with environmental perturbations. The relative intensities of the two peaks of *v*(P-OFe) at 1008 cm^−1^ and *v*(P-O) at 1128 cm^−1^ were similar in the previous theoretical DFT study by Yang *et al*.^30^ and the experimental study by Kubicki *et al*.^12^, but the intensity of *v*(P-OFe) at 1012 cm^−1^ was significantly higher than that of *v*(P-O) at 1128 cm^−1^ in this study. This result indicates that bridging complexes between two Gt nanorods were formed. However, the vibration mode and frequency would differ in the bridging complex; thus, further DFT calculations should be performed. Acelas *et al*. calculated the relative Gibbs free energy using DFT, and the BBH_1_ complex was more favourable than the monodentate complex under acidic and intermediate pH conditions^13^. However, this study and a previous study by Arroyeve *et al*.^29^ showed similar spectra for nanorod-shaped Gt compared with the spectrum from the study by Kubicki *et al*.^29^, but there may be a bridging complex with more thermodynamically favourable properties than those of the monodentate complex. Only 4 schemes were described in this study, but an additional experiment employed DW and phosphate addition after repeated hydration and dehydration. As a result, a background resulting from the slow water seepage velocity into the nanopores of Gt was constantly observed with DW addition, while the increase in background absorbance gradually decreased with 3 repetitions of phosphate addition, which indicates the blocking of nanopores by phosphate adsorption. In addition, the IA of P-Gt showed no fluctuations with environmental perturbations, which implies that a more stable complex of phosphate was present or that the complex was present in a less accessible space.

The swelling effect on P-Mh is explained by the zeta potential change due to phosphate adsorption. As discussed above, BBH_0_ and BBH_1_ complexes were dominant on the Mh surface. Antelo *et al*. reported the effect of arsenate and phosphate complexes on the zeta potential of a surface^32^. As a result, the formation of BBH_0_ and BBH_1_ complexes changed the surface charge from positive to negative; thus, the repulsive force of negatively charged phosphate-adsorbed Mh in bidentate form caused the swelling effect during phosphate adsorption. In addition, DW bridging decreased the ionic strength and thickness of the diffuse double layer; thus, the distance between Mh NPs was increased. Unlike DW bridging in S-4, P addition in S-3 showed a smaller decrease in IA for the Mh film. It was presumed that the experimental setup experienced dehydration over time and the water molecules on the binary films were fully dried after 240 min. For that reason, the ionic strength of the films before phosphate addition was higher than that of the newly injected phosphate solution, which increased the thickness of the diffuse double layer.

Shrinking and swelling effects could explain the opposite patterns after phosphate addition in S-1 and S-3. Dehydration was employed in S-1 after single adsorption and DW bridging, which was caused by the blocking of nanopores. No dehydration was applied in S-3. The spectrum for phosphate addition in S-1 was recorded 720 min after the initial phosphate input, while the spectrum of S-3 was recorded after 150 min. The results imply that the nanopores of Gt in S-1 were closed by repeated dehydration with phosphate; thus, only the Mh surface was available for additional phosphate. In contrast, the phosphate in the nanopores of Gt was not completely equilibrated, and the nanopores were not closed by dehydration. In addition, the swelling effect increased the IA of P-Gt in the evanescent wave region. The IA of P-Mh was presumed to be caused by compensation for the increased P-Mh on the surface over time and the decreased Mh film in S-3.

Both shrinking and swelling properties are critical factors in addressing phosphate adsorption on films using *in situ* ATR-FTIR. However, no studies have yet addressed these effects in iron (hydr)oxides, to the best of our knowledge, and these effects would cause misinterpretation of the adsorption spectra. For this reason, shrinking and swelling properties should be fully examined in NP studies using *in situ* ATR-FTIR, which measures the partial volume of samples.

### Environmental implications

In this study, the preferential adsorption of phosphate on binary Gt and Mh surfaces was characterized. The experimental procedure was developed to measure preferential adsorption using *in situ* ATR-FTIR, and the spectral evolution was recorded to identify phosphate adsorption on a single sorbent and binary sorbents and the effect of drying on preferential adsorption. Based on FE-SEM/EDS analysis and LLS regression of the recorded spectra, phosphate was preferentially adsorbed onto the Mh surface rather than the Gt surface, confirming the previous batch experiment. The distinctive aggregation properties of the two sorbents mainly caused preferential adsorption because the adsorption sites on the nanopores of Gt were decreased by side-by-side aggregation, while the adsorption sites on spherical Mh were unchanged. The distinctive properties showed the shrinking and swelling effect of iron (hydr)oxide NPs and caused fluctuations in adsorbed phosphate. In addition, there is the possibility of bridging complexes on Gt, which would cause side-by-side aggregation, and repeated hydration and dehydration conditions made the nanopores of Gt inaccessible and led to stable phosphate complexes under environmental perturbation.

The experimental setup in this study was not equivalent to reactions in the real environment, but it is still reasonable to extrapolate the results to the real environment. Phosphate availability is the most important concern for fertilization in agriculture and eutrophication in the water environment. If phosphate on Gt forms bridging complexes by aggregating and blocking nanopores, this behaviour could explain the low phosphate bioavailability in the real soil environment, where Gt is the most common iron oxide in soils worldwide. In contrast, bidentate complexes of phosphate on Mh would increase the colloidal properties of Mh, which is easily leachable to the water system upon environmental perturbation.

There are numerous limitations and shortcomings in the experimental procedure proposed in this study. In the real soil environment, soil is a mixture of uncountable components, and various chemicals interact with the numerous surfaces of the soil components. It is easy to examine the total concentration of a target chemical in a soil, but it is difficult to analyse the target chemical adsorption on a specific surface among soil components at the water-soil interface, and it is also difficult to understand the adsorption mechanism when various surfaces are available for adsorption. The experimental procedure proposed in this study would enable us to observe preferential adsorption on multi iron (hydr)oxide sorbents and identify actual preferential adsorption at the interface and in real time. The experimental procedure is still a simplification of the reaction in the real world, but we believe that the current bottom-up approach will eventually reveal unknown mechanisms in the soil environment.

## Materials and Methods

### Physicochemical characterization and Langmuir isotherm

Rod-shaped nanoGt (Cat. No. US3162) was purchased from US Research Nanomaterials (USA), and sphere-shaped nanoMh (Cat. No. 544884) was purchased from Sigma-Aldrich (USA). For characterization of the crystal structure, XRD was applied using a D8 Advance (Bruker, Germany) and AMCSD^33^. The point of net zero charge, electrical conductivity, pH and SA were measured, and HR-TEM was used for morphology and elemental concentration characterization. All physicochemical characteristics and the adsorption capacity of the two sorbents using the Langmuir isotherm were previously reported (Table S1)^5,21^. Sedimentation was briefly evaluated by mixing 0.25 g of Gt and Mh with various phosphate concentrations (0, 0.5 mM and 5 mM) and agitating for 10 min, and part of the sample was transferred to a plastic cuvette to obtain an image of sedimentation. The difference in adsorption capacity (*Q*) between the suspended NPs in the agitated solution and the film was measured. A total of 0.1 g of iron (hydr)oxide was mixed with 20 mL of DW in a glass vial and dried at 50 °C for 3 days for film formation. Then, 20 mL of 1 mM phosphate solution was added to the glass vial with the film. After equilibration for 2 days, the supernatant was sampled, and the concentrations of phosphorous and iron were measured using inductively coupled plasma optical emission spectrometry (ICP-OES) (Icap 7200, Thermo, USA).

### Experimental schemes

Four experimental schemes were designed and conducted: S-1: Sequential single adsorption on two sorbents, drying, bridging the binary sorbents with DW, drying and phosphate addition; S-2: Saturation of binary sorbents with DW for 5 min, simultaneous adsorption on binary sorbents and adding DW to prevent dehydration; S-3: Saturation of binary sorbents with DW for 60 min, simultaneous adsorption on binary sorbents, and phosphate addition; and S-4: Saturation of binary sorbents with DW for 60 min, overlapping single adsorption on binary sorbents, bridging with DW without dehydration, drying and DW addition. The four schemes were designed and conducted to evaluate the effect of phosphate addition type (single or simultaneous) and hydration conditions (dried or saturated) on the adsorption of phosphate on binary sorbents. Detailed schemes are illustrated in Fig. 1.

### *In situ* ATR-FTIR measurements

Gt or Mh was dispersed in DW at 1 g L^−1^, and 0.75 mL of dispersions of Gt and Mh were placed side by side on the ZnSe ATR crystal (77 × 8 × 2 mm and 45° cut edges, Piketech, USA) to form binary sorbent layers. The ATR plate was stored at room temperature (controlled to 25 ± 2 °C) and dried for at least 24 h before the experiment. A phosphate solution containing 1 mM or 10 mM NaH_2_PO_4_ and 10 mM NaCl was prepared, and the pH of the phosphate solution was adjusted to 4.0 with 0.1 M HCl or NaOH solution. The phosphate adsorption at the interface and the spectra of dried samples were measured for the four experimental schemes described above. For binary film formation, two films on a single ZnSe crystal were prepared. There was an approximately 9~12 mm gap between the two films. Thus, 1.5 mL of DW was added instead of 1 mL to form the bridge between the sorbents and to extend the reaction time. In detail, bridging is defined as the first binary adsorption to make the bridge, and addition is defined as the sequential input of DW or P solution after bridging. All spectra were collected by an IR Tracer-100 (Shimadzu, Japan) equipped with a deuterated triglycine sulfate detector. Thirty-two scans were co-added at a resolution of 4 cm^−1^ under Ar purging conditions. Happ-Genzel apodization was applied, and the aperture was set to 3. The time interval between each of the spectra was approximately 40 sec. All spectra were collected by IR solution software (Shimadzu, Japan), and spectral processing was conducted using Essential FTIR software (Operant LLC, USA). All analytical chemicals were purchased from Sigma-Aldrich (USA) at a high purity grade.

### Spectral analysis

The time series of measured spectra in each scheme was adjusted by subtracting the initial spectra. Two types of initial conditions were applied: reacting phosphate solution on films with and without DW pre-saturation to infer the effect of dehydration on the swelling properties of iron (hydr)oxides. The stability of the films was evaluated by calculating the change in PA in the dried samples, which was 870-760 cm^−1^ for Gt and 760-670 cm^−1^ for Mh, where distinctive peaks were observed.

The measured spectra in each experiment showed dynamic changes during perturbations. 2D-COS is a powerful and versatile technique to separate complex overlapping signals; thus, 2D-COS was applied to identify the positions and sequence of bands using 2D Shige software by Shigeaki Mortia (Kwansei-Gakuin University, Japan). Synchronous and asynchronous spectra for different times and experimental schemes were calculated to identify the band position and sequence of band appearance over time. In synchronous spectra, autopeaks cause changes in peak intensity over time, while crosspeaks indicate the time perturbation of two different peaks.

The measured spectra are a combination of numerous signals, and it is essential to characterize the individual fractions of the components; thus, LLS regression with the Levenberg-Marquardt algorithm was employed using SigmaPlot 10 (Systat, USA). The components of fitting included the spectra of single P-Gt and P-Mh, water background, aqueous phosphate at pH 4, precipitated phosphate with 10 mM NaCl, Gt film and Mh film. All calculations employed 50 fits with 2,000 iterations, and adj. r^2^ was calculated to show the goodness of fit.

## Supplementary information

Supporting information

## References

[1] Elser JJ (2007). Global analysis of nitrogen and phosphorus limitation of primary producers in freshwater, marine and terrestrial ecosystems. Ecol. Lett..

[2] Barberis E (1995). European soils overfertilized with phosphorus: Part 1. Basic properties. Fertil. Res..

[3] Han J, Ro H-M, Cho KH, Kim K-W (2016). Fluxes of nutrients and trace metals across the sediment-water interface controlled by sediment-capping agents: bentonite and sand. Environ. Monit. Assess..

[4] Kubicki JDJ, Kwon KD, Paul KW, Sparks DL (2007). Surface complex structures modelled with quantum chemical calculations: Carbonate, phosphate, sulphate, arsenate and arsenite. Eur. J. Soil Sci..

[5] Han, J. & Ro, H. Interpreting competitive adsorption of arsenate and phosphate on nanosized iron (hydr) oxides: effects of pH and surface loading. *Environ. Sci. Pollut. Res.* **25**, 28572–28582 (2018). 10.1007/s11356-018-2897-y 30091077

[6] Parikh SJ, Mukome FND, Zhang X (2014). ATR–FTIR spectroscopic evidence for biomolecular phosphorus and carboxyl groups facilitating bacterial adhesion to iron oxides. Colloids Surf., B.

[7] Zheng TT, Sun ZX, Yang XF, Holmgren A (2012). Sorption of phosphate onto mesoporous γ-alumina studied with *in-situ* ATR-FTIR spectroscopy. Chem. Cent. J..

[8] Elzinga EJ, Huang J-H, Chorover J, Kretzschmar R (2012). ATR-FTIR spectroscopy study of the influence of pH and contact time on the adhesion of Shewanella putrefaciens bacterial cells to the surface of hematite. Environ. Sci. Technol..

[9] Atkinson RJ, Parfitt RL, Smart RSC (1974). Infra-red study of phosphate adsorption on goethite. J. Chem. Soc. Faraday Trans. 1 Phys. Chem. Condens. Phases.

[10] Tofan-Lazar J, Al-Abadleh HAH (2012). Kinetic ATR-FTIR Studies on Phosphate Adsorption on Iron (Oxyhydr)oxides in the Absence and Presence of Surface Arsenic: Molecular-Level Insights into the Ligand Exchange Mechanism. J. Phys. Chem. A.

[11] Elzinga EJ, Sparks DL (2007). Phosphate adsorption onto hematite: An *in situ* ATR-FTIR investigation of the effects of pH and loading level on the mode of phosphate surface complexation. J. Colloid Interface Sci..

[12] Kubicki J, Paul K, Kabalan L, Zhu Q (2012). ATR–FTIR and Density Functional Theory Study of the Structures, Energetics, and Vibrational Spectra of Phosphate Adsorbed onto Goethite. Langmuir.

[13] Acelas NY, Mejia SM, Mondragón F, Flórez E (2013). Density functional theory characterization of phosphate and sulfate adsorption on Fe-(hydr)oxide: Reactivity, pH effect, estimation of Gibbs free energies, and topological analysis of hydrogen bonds. Comput. Theor. Chem..

[14] Waiman CV, Avena MJ, Regazzoni AE, Zanini GP (2013). A real time *in situ* ATR-FTIR spectroscopic study of glyphosate desorption from goethite as induced by phosphate adsorption: Effect of surface coverage. J. Colloid Interface Sci..

[15] Arai Y, Sparks DL (2001). ATR–FTIR Spectroscopic Investigation on Phosphate Adsorption Mechanisms at the Ferrihydrite–Water Interface. J. Colloid Interface Sci..

[16] Carabante I, Grahn M (2010). *In situ* ATR–FTIR studies on the competitive adsorption of arsenate and phosphate on ferrihydrite. J. Colloid Interface Sci..

[17] Lindegren M, Persson P (2009). Competitive adsorption between phosphate and carboxylic acids: Quantitative effects and molecular mechanisms. Eur. J. Soil Sci..

[18] Rahnemaie R, Hiemstra T, van Riemsdijk WH (2007). Carbonate adsorption on goethite in competition with phosphate. J. Colloid Interface Sci..

[19] Abdala DB, Northrup PA, Arai Y, Sparks DL (2015). Surface loading effects on orthophosphate surface complexation at the goethite/water interface as examined by extended X-ray Absorption Fine Structure (EXAFS) spectroscopy. J. Colloid Interface Sci..

[20] Daou, T. J. *et al*. Phosphate Adsorption Properties of Magnetite-Based Nanoparticles Phosphate Adsorption Properties of Magnetite-Based Nanoparticles. **19**, 4494–4505 (2007).

[21] Han J, Ro H (2018). Identification of Bernalite Transformation and Tridentate Arsenate Complex at Nano- goethite under Effects of Drying, pH and Surface Loading. Sci. Rep..

[22] Yang H, Lu R, Downs RT, Costin G (2006). Goethite, α-FeO(OH), from single-crystal data. Acta Crystallogr. Sect. E Struct. Reports Online.

[23] Gao Y, Mucci A (2001). Acid base reactions, phosphate and arsenate complexation, and their competitive adsorption at the surface of goethite in 0.7 M NaCl solution. Geochim. Cosmochim. Acta.

[24] Manning BA, Goldberg S (1996). Modeling Competitive Adsorption of Arsenate with Phosphate and Molybdate on Oxide. Minerals. Soil Sci. Soc. Am. J..

[25] Boukemara L, Boukhalfa C, Reinert L, Duclaux L (2016). Characterization of phosphate adsorption on goethite macroscopic and spectroscopic analyses. J. Mater. Environ. Sci..

[26] Geelhoed JS, Hiemstra T, Van Riemsdijk WH (1997). Phosphate and sulfate adsorption on goethite: Single anion and competitive adsorption. Geochim. Cosmochim. Acta.

[27] Dickson D, Liu G, Cai Y (2017). Adsorption kinetics and isotherms of arsenite and arsenate on hematite nanoparticles and aggregates. J. Environ. Manage..

[28] Persson P, Nilsson N, Sjöberg S (1996). Structure and Bonding of Orthophosphate Ions at the Iron Oxide-Aqueous Interface. J. Colloid Interface Sci..

[29] Arroyave JM, Puccia V, Zanini GP, Avena MJ (2018). Surface speciation of phosphate on goethite as seen by InfraRed Surface Titrations (IRST). Spectrochim. Acta - Part A Mol. Biomol. Spectrosc..

[30] Yang, Y., Wang, S., Xu, Y., Zheng, B. & Liu, J. Molecular-Scale Study of Aspartate Adsorption on Goethite and Competition with Phosphate. *Environ. Sci. Technol*. **50**, acs.est. 5b05450 (2016). 10.1021/acs.est.5b05450 26870876

[31] Farrell J (2017). Tridentate arsenate complexation with ferric hydroxide and its effect on the kinetics of arsenate adsorption and desorption. Chemosphere.

[32] Antelo J, Avena M, Fiol S, López R, Arce F (2005). Effects of pH and ionic strength on the adsorption of phosphate and arsenate at the goethite-water interface. J. Colloid Interface Sci..

[33] Downs R, Hall-Wallace M (2003). The American Mineralogist crystal structure database. Am. Mineral..

