# Supplementary material for: Characterizing Preferential Adsorption of Phosphate on Binary Sorbents of Goethite and Maghaemite using in situ ATR-FTIR and 2D Correlation Spectroscopy
Source: Sci Rep. 2019 Apr 16;9:6130. doi: 10.1038/s41598-019-42575-2 (PMC6467979; doi:10.1038/s41598-019-42575-2)
Supplement: Supplementary file 1 — Supporting information [file 41598_2019_42575_MOESM1_ESM.docx]

Supporting Information

Characterizing Preferential Adsorption of Phosphate on Binary Sorbents of Goethite and Maghaemite using *in situ* ATR-FTIR and 2D Correlation Spectroscopy

Junho Han^1^, Hee-Myong Ro^1*^

^1^Department of Agricultural Biotechnology and Research Institute of Agriculture and Life Sciences, Seoul National University, Seoul, 08826 Republic of Korea

*To whom correspondence should be addressed;

Hee-Myong Ro

Phone: 82-2-880-4645

Fax: 82-2-873-3122

Email: hmro@snu.ac.kr

Number of Pages: 13

Number of Tables: 4

Number of Figures: 8

**Table S1** Physicochemical characteristics of goethite and maghaemite.

| Iron Oxide | Formula | Manufacturer  (Catalog #) | Shape | Crystal  Structure | pH | EC | PZC | Measured  SA  (m^2^ g^-1^) | Calculated  SA  (m^2^ g^-1^) | Cell Volume  (nm^3^) | Proposed  Size (nm) | Measured  Size  (nm) |
| --- | --- | --- | --- | --- | --- | --- | --- | --- | --- | --- | --- | --- |
| Goethite | FeOOH | US-Nano  (US3162) | Rod | isometric | 5.7 | 0.13 | 5.7 | 84.0 | 113.1 | 0.1386 | 50x10 | 50.3x10.8  (*n*=151) |
| Maghaemite | Fe_2.67_O_4_ | Sigma-Aldrich  (544884) | Spherical | isometric | 5.1 | 0.14 | 4.7 | 35.6 | 23.0 | 0.5825 | 30 | 53.7  (*n*=116) |

*pH* proton concentration, *EC* electric conductivity (μS cm^-1^), PZC point of zero charge, SA surface area (m_2_ g^-1^)

pH and EC were measured at 1:200 (g:mL).

PZC and SA were measured by the drift method and N_2_-BET method at 77K, respectively.

**Table S2** Integrated peak areas between two distinctive peaks of goethite (840-760 cm^-1^) and maghaemite (760-670 cm^-1^) to evaluate the initial condition of binary films and the difference before and after adsorption experiment. The ATR-FTIR spectra after dehydration were employed for the ratio calculation.

| Recoreded spectrum | Scheme | Peak area at  840-760 cm^-1^ | Peak area at  760-670 cm^-1^ | Ratio between  two peak areas | Ratio between before and after adsorption |
| --- | --- | --- | --- | --- | --- |
| Single film | Goethite | 36.48 | 20.59 | 1.772 | - |
|  | Maghaemite | 8.762 | 54.44 | 0.161 | - |
| Binary films  before adsorption | S1 | 27.26 | 41.65 | 0.654 | - |
|  | S2 | 27.45 | 39.06 | 0.703 | - |
|  | S3 | 27.78 | 41.77 | 0.665 | - |
|  | S4 | 27.24 | 39.21 | 0.695 | - |
| Binary films  after adsorption | S1 | 28.03 | 44.47 | 0.630 | 0.963 |
|  | S2 | 26.89 | 38.74 | 0.694 | 0.988 |
|  | S3 | 32.06 | 41.81 | 0.767 | 1.152 |
|  | S4 | 29.11 | 40.36 | 0.721 | 1.038 |

**Table S3** Correlation matrix of identified peaks during the single goethite adsorption by 2D-COS analysis.

|  | 863 cm^-1^ | 885 cm^-1^ | 915 cm^-1^ | 975 cm^-1^ | 1012 cm^-1^ | 1075 cm^-1^ | 1125 cm^-1^ | 1180 cm^-1^ | 1295 cm^-1^ |
| --- | --- | --- | --- | --- | --- | --- | --- | --- | --- |
| 863 cm^-1^ | 1 | 0.983^*^ | 0.986^*^ | 0.886^*^ | 0.878^*^ | 0.767 | 0.857^*^ | 0.880^*^ | 0.855^*^ |
| 885 cm^-1^ |  | 1 | 0.969^*^ | 0.928^*^ | 0.916^*^ | 0.824 | 0.897^*^ | 0.907^*^ | 0.894^*^ |
| 915 cm^-1^ |  |  | 1 | 0.840^*^ | 0.830 | 0.688 | 0.793 | 0.825 | 0.789 |
| 975 cm^-1^ |  |  |  | 1 | 0.994^*^ | 0.957^*^ | 0.989^*^ | 0.914^*^ | 0.957^*^ |
| 1012 cm^-1^ |  |  |  |  | 1 | 0.944^*^ | 0.981^*^ | 0.875^*^ | 0.933^*^ |
| 1075 cm^-1^ |  |  |  |  |  | 1 | 0.977^*^ | 0.914^*^ | 0.971^*^ |
| 1125 cm^-1^ |  |  |  |  |  |  | 1 | 0.920^*^ | 0.967^*^ |
| 1180 cm^-1^ |  |  |  |  |  |  |  | 1 | 0.972^*^ |
| 1295 cm^-1^ |  |  |  |  |  |  |  |  | 1 |

**Table S4** Correlation matrix of identified peaks during the single maghaemite adsorption by 2D-COS analysis.

|  | 950 cm^-1^ | 1026 cm^-1^ | 1069 cm^-1^ | 1078 cm^-1^ | 1094 cm^-1^ | 1155 cm^-1^ | 1358 cm^-1^ |
| --- | --- | --- | --- | --- | --- | --- | --- |
| 950 cm^-1^ | 1 | 0.997^*^ | 0.999^*^ | 0.998^*^ | 0.998^*^ | 0.982^*^ | -0.974^*^ |
| 1026 cm^-1^ |  | 1 | 0.998^*^ | 0.998^*^ | 0.999^*^ | 0.966^*^ | -0.985^*^ |
| 1069 cm^-1^ |  |  | 1 | 1.000^*^ | 0.999^*^ | 0.979^*^ | -0.983^*^ |
| 1078 cm^-1^ |  |  |  | 1 | 1.000^*^ | 0.977^*^ | -0.985^*^ |
| 1094 cm^-1^ |  |  |  |  | 1 | 0.972^*^ | -0.986^*^ |
| 1155 cm^-1^ |  |  |  |  |  | 1 | -0.936^*^ |
| 1358 cm^-1^ |  |  |  |  |  |  | 1 |


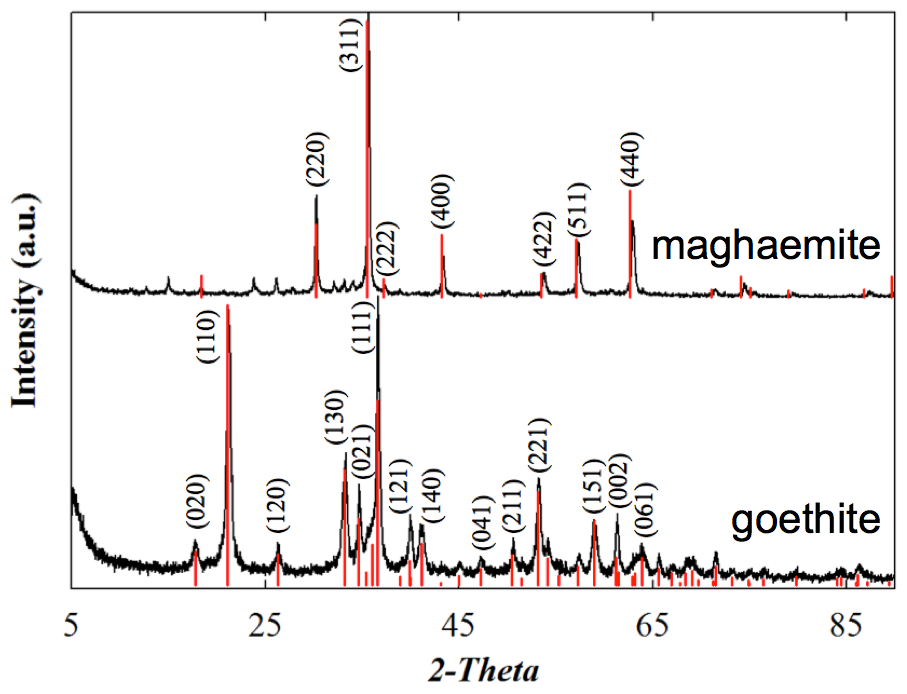


**Fig. S1** X-ray diffraction pattern of goethite and maghaemite. All peaks are indexed to the goethite and maghaemite phase. The red bars indicate the reference position (red bar) from the American mineralogist crystal structure database.


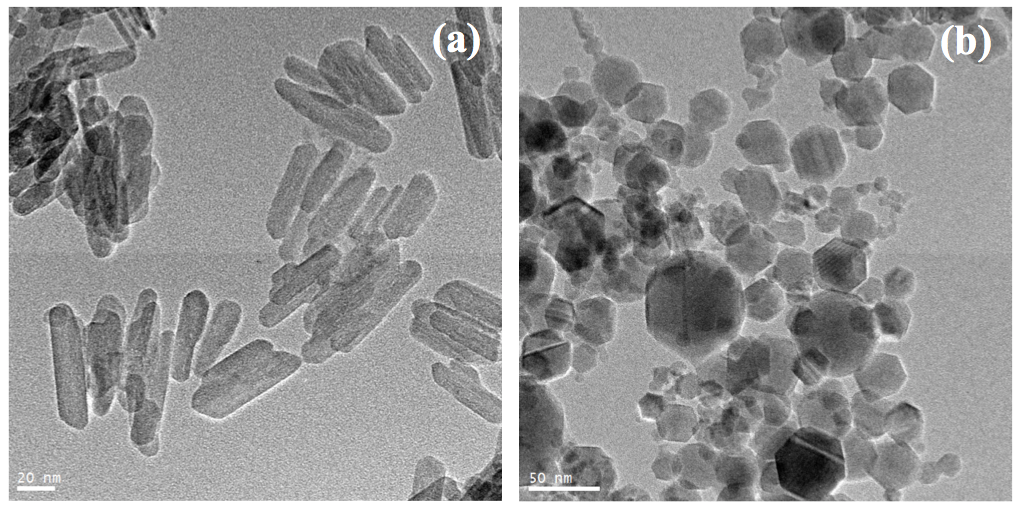


**Fig. S2** HR-TEM image of nanosized goethite (a) and maghaemite (b).


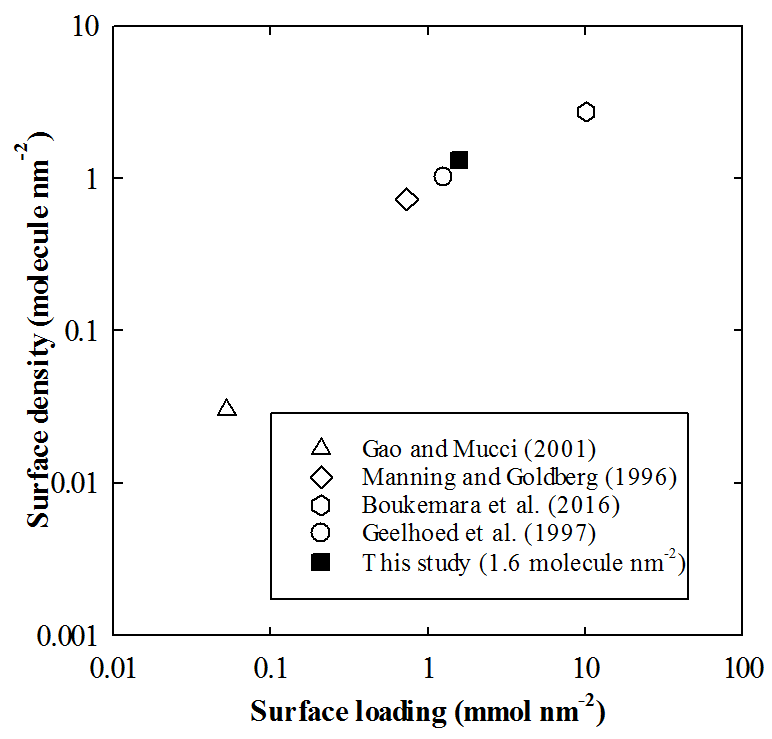


**Fig. S3** Scatter plot between the surface loading (molecule nm^-2^) and surface density (molecule nm^-2^) on the goethite from the previous literature (triangle, diamond, hexagon and circle) and this study (rectangle) at pH. The axis was log-scaled for better illustration.


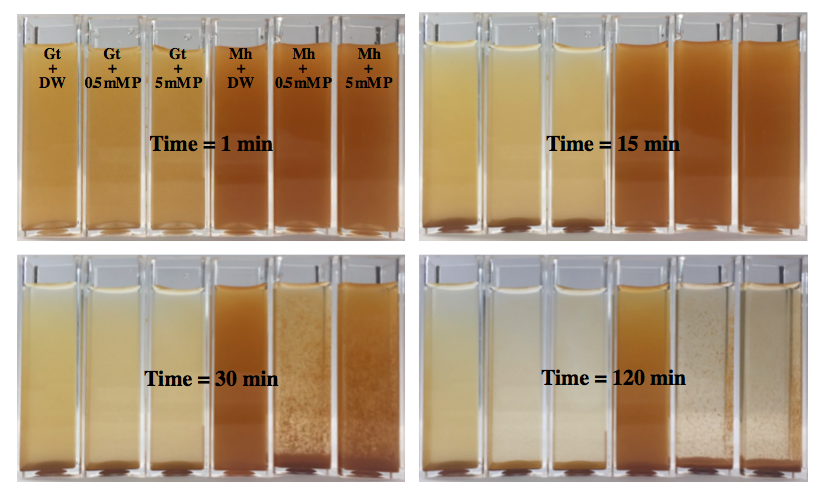


**Fig. S4** Sedimentation photographs of nanosized goethite (Gt) and maghaemite (Mh) at different phosphate concentrations (0, 0.5 and 5 mM) after standing for 1, 15, 30 and 120 min. DW is the abbreviation of distilled water.

**
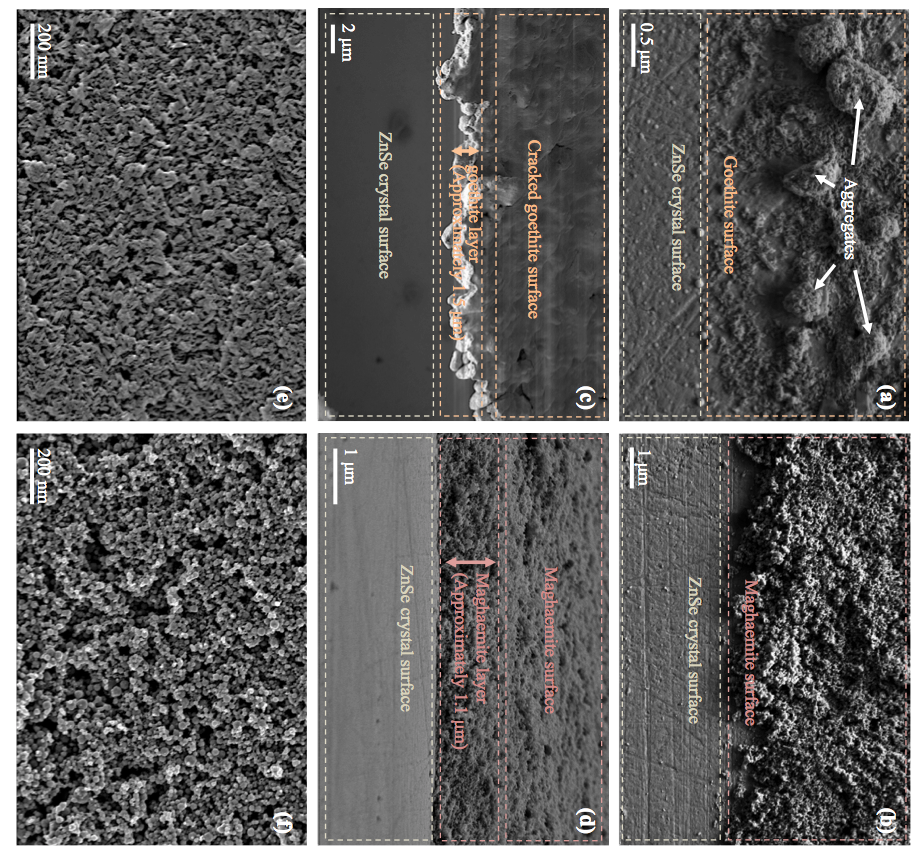
**

**Fig. S5** Micro-scale image of the goethite surface (a) and maghaemite surface (b) on the ZnSe crystal, 20-degree tilted image of goethite layer (c) and maghaemite surface (d) on ZnSe crystal by SE2 mode under 25 kV using FE-SEM, and nanoscale image of the goethite (e) and maghaemite film (f) by InLens mode under 2 kV.


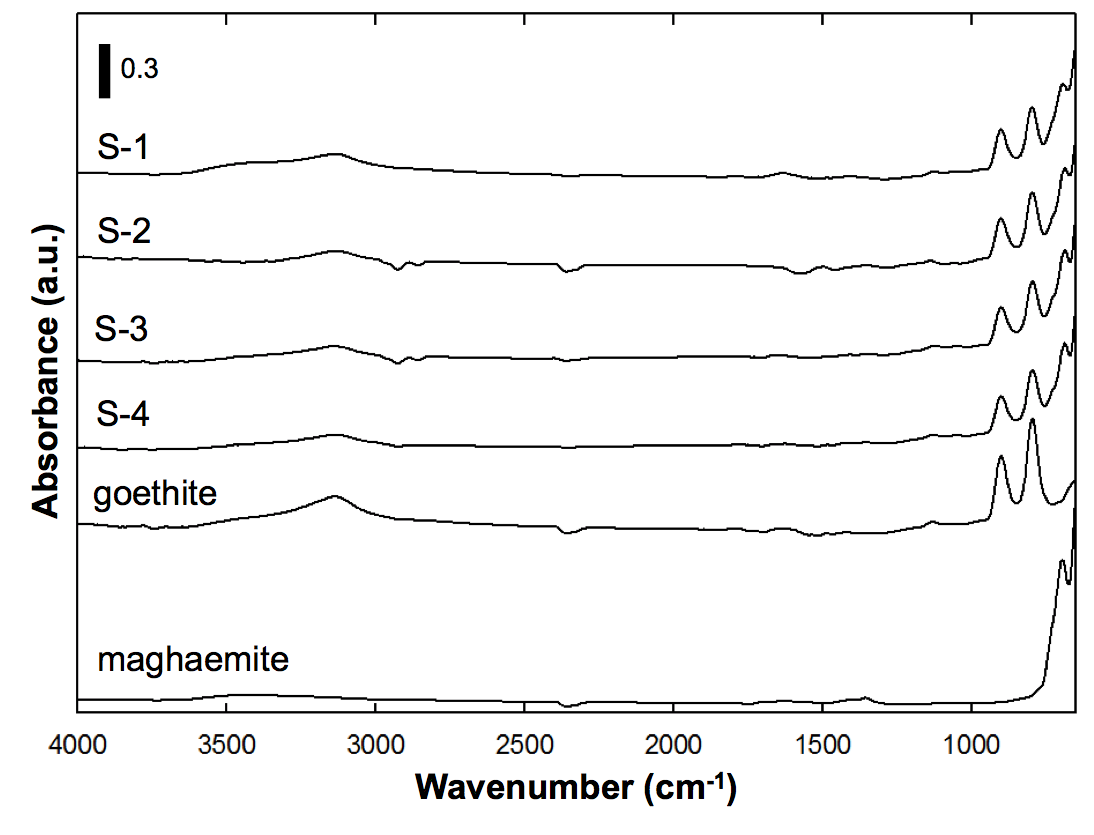


**Fig. S6** ATR-FTIR spectra of single film and binary films used for scheme 1 to 4.


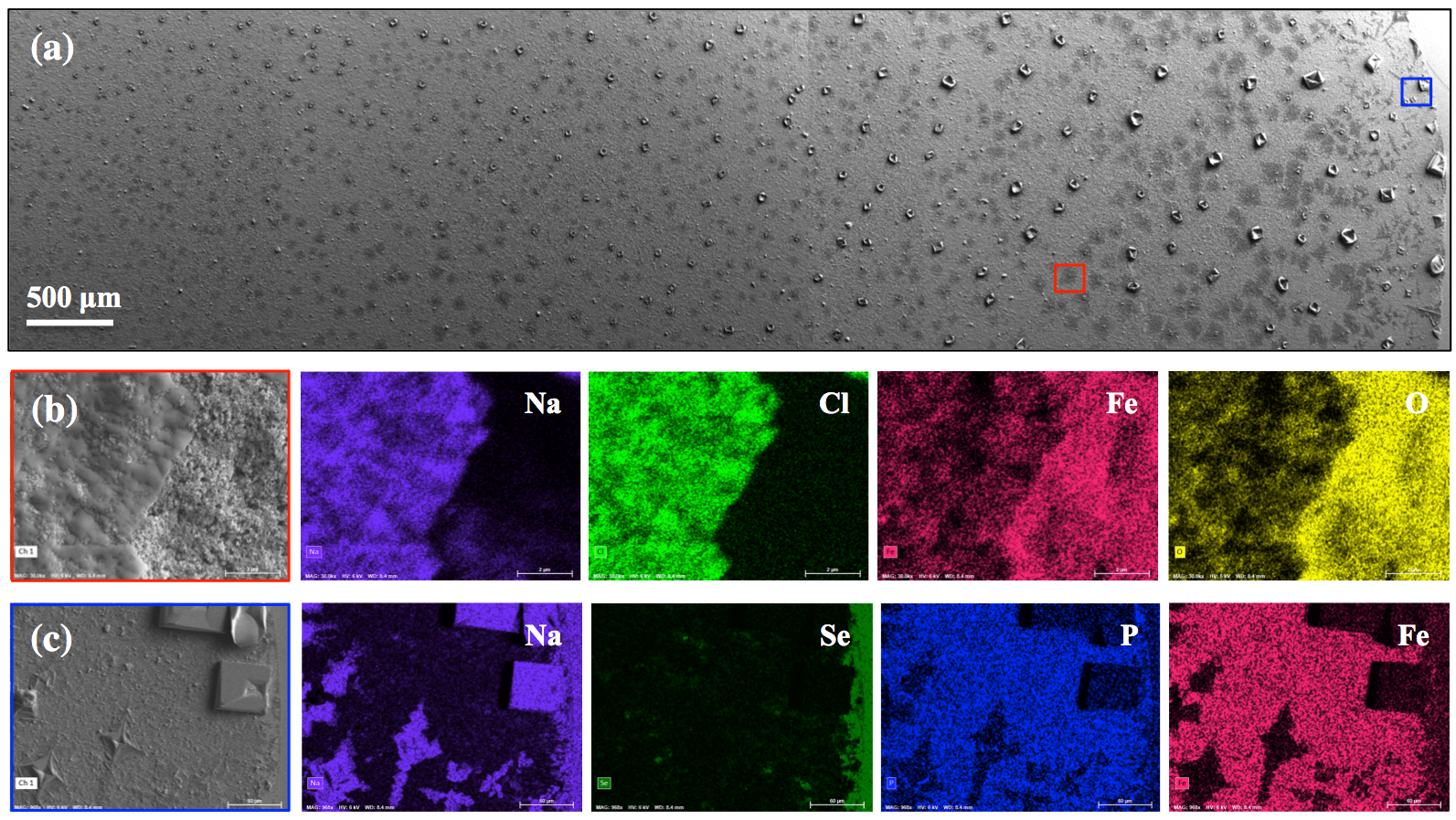


**Fig. S7** Stitched image of FE-SEM at 100 x magnification using SE2 mode (a), mapping image of FE-SEM-EDS at 30,000 x magnification at dark spot (b) and crystal structure (c). The right side is the marginal part of the maghaemite layer, and the left side is the direction to the center part of layer. The red and blue boxes in (a) indicate the location of the mapping image (b) and (c), respectively.


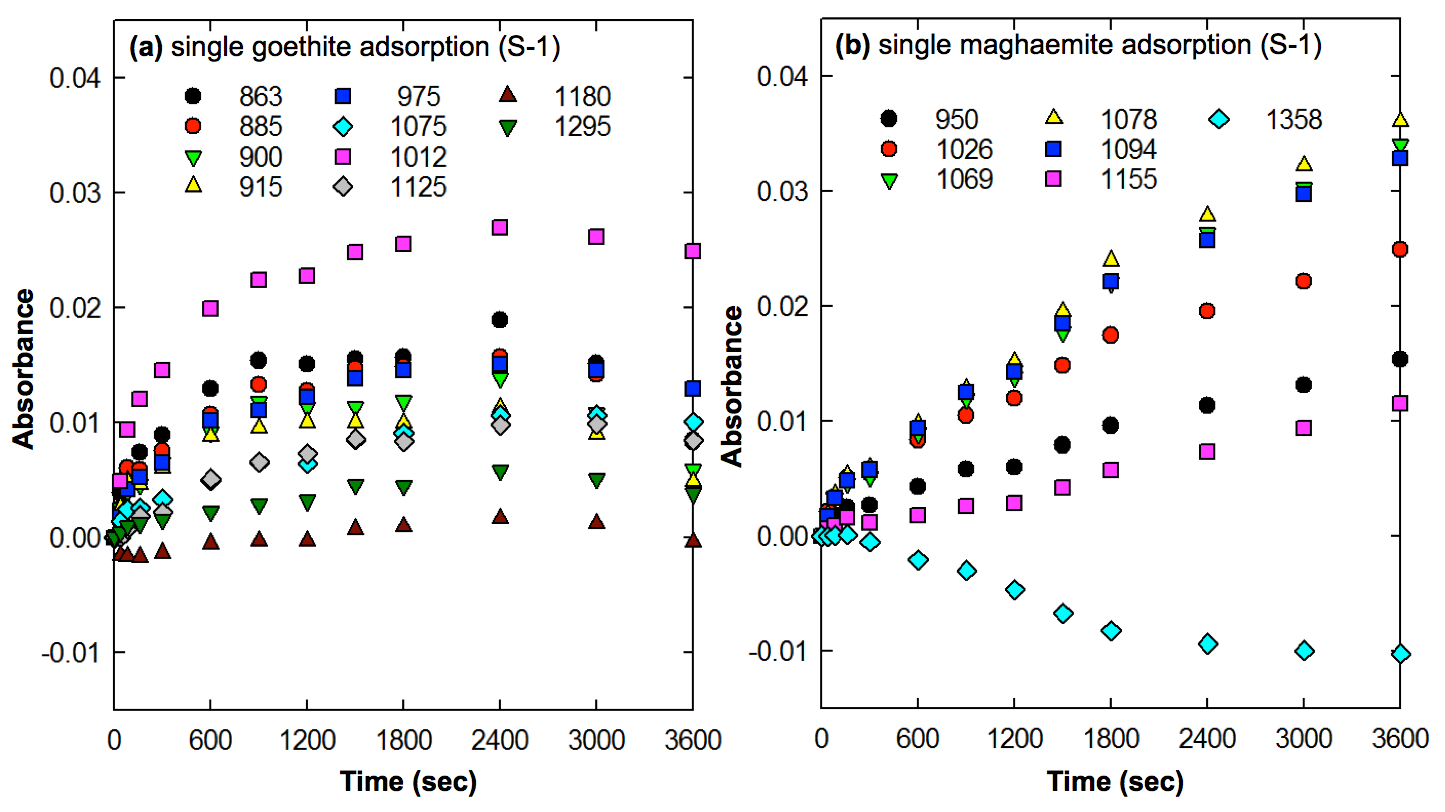


**Fig. S8** Absorbance change of single adsorption experiment. The numbers indicate the wavenumber identified by the 2D-COS analysis.
